# Safety and Efficacy of Manual and Automated Abdominal Colonic Massage for Chronic Constipation: A Systematic Review and Meta-Analysis

**DOI:** 10.14309/ctg.0000000000001027

**Published:** 2026-04-01

**Authors:** M. Eugenia Delgado, Josep Benet, Immaculada Herrero-Fresneda

**Affiliations:** 1 Department of Pharmacology, Toxicology and Therapeutic Chemistry, Toxicology Section, Faculty of Pharmacy and Food Sciences, University of Barcelona, Barcelona, Spain;; 2 Independent Researcher in Biomedical Research Methodology, Preventive Medicine and Public Health, Barcelona, Spain;; 3 USMIMA S.L, Barcelona, Spain;; 4 Faculty of Medicine, University of Vic, Central University of Catalonia, Barcelona, Spain.

**Keywords:** abdominal colonic massage, manual, automatic, chronic constipation

## Abstract

**INTRODUCTION:**

Abdominal colonic massage shows benefits against chronic constipation (CC). While both manual and automated versions of this therapy show potential, there is currently no comprehensive synthesis comparing their effectiveness and safety.

**METHODS:**

Studies on manual and device-assisted abdominal massage for CC were identified from major bibliographic databases (1990–2025). Risk of bias was assessed. Outcomes included bowel movements per week (BM/w), quality of life (QoL), and adverse events. Meta-analyses were stratified by patient profile and intervention type. Where available, control data were extracted and compiled in a third group compared with manual and automated intervention through POST-PRE analysis of variance statistical synthesis.

**RESULTS:**

Of 1,352 records screened, 54 studies met inclusion criteria, 35 were included in the meta-analysis (1965 patients). No study was judged at high risk of bias. Meta-analysis showed significant improved BM/w and QoL after both manual (*p*_*BM/w&QoL*_ < 0.00001) and device-assisted (*p*_*BM/w*_ = 0.0006; *p*_*QoL*_ < 0.00001). Stratified analyses showed consistent effect estimates in functional constipation/chronic idiopathic constipation across intervention types, whereas neurogenic bowel dysfunction meta-analysis was not feasible because of limited studies. No significant difference was observed between manual or device-assisted outcomes in any subgroup. Sensitivity analyses confirmed the robustness of results. Statistical synthesis confirmed both manual and automated interventions outperformed controls (BM/w: x5.9–x7.6; *P* = .0006; QoL: x2.4–x4.1; *P* < 0.005) with no significant difference between them. Adverse events were rare (6.3%), mild, and self-limiting.

**DISCUSSION:**

Abdominal massage is a safe and effective treatment for CC from both neurogenic bowel dysfunction and functional constipation/chronic idiopathic constipation origin. The automated version is equal or more effective than the manual, making it a strong candidate for inclusion in regular clinical practice.

## Graphical abstract

**Figure:**
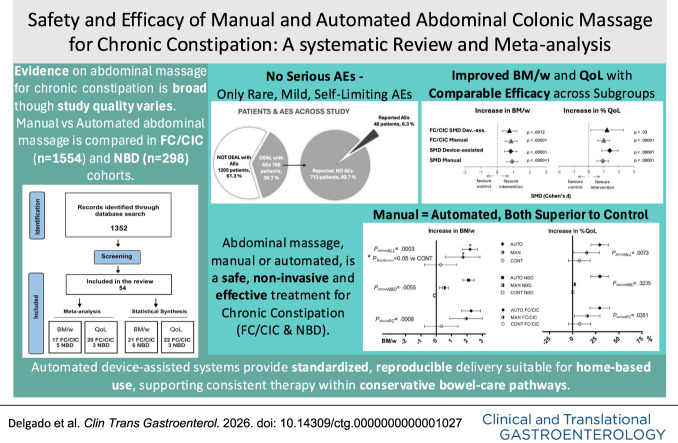

## INTRODUCTION

Chronic constipation (CC), a common condition in Western society, remains a widely recognized unmet therapeutic need (1). It can cause significant physical discomfort, psychological distress, and social impairment, profoundly affecting quality of life (QoL) and placing a considerable burden on healthcare systems (2,3).

Effective management requires a multifaceted approach because no single intervention addresses all symptoms or underlying causes (4). Treatments are commonly structured using a stepwise model (5), which escalates care based on symptom severity and response. This approach provides immediate relief and symptom management, even if the underlying cause is not fully understood at the outset. However, long-term follow-up studies reveal that nearly 50% of adults remain symptomatic even after 5 years of treatment (6,7), underscoring the persistent and refractory nature of the condition.

Abdominal colonic massage is widely recommended as a noninvasive therapy for CC, aiming to support and stimulate natural peristalsis and improve bowel motility (8). Clinical evidence suggests it may increase evacuation frequency and symptom relief in patients with functional or neurogenic constipation, alongside improved autonomy and self-management (9–12). However, even with a standardized protocol, reproducibility remains a challenge because of variability in practitioner technique, applied pressure, and individual execution. Moreover, practical implementation is constrained by the need for daily application, consistent timing, adequate pressure and proper technique, requirements that are particularly difficult for individuals with limited mobility, reduced manual dexterity, or lack of caregiver support (8,13).

Automated colonic massage devices aim to overcome these drawbacks by providing standardized compression patterns and forces, readily available for daily use. Previous clinical trials have shown that such medical devices applying abdominal massage are safe, well tolerated, and effective in improving bowel function in individuals with chronic or neurogenic constipation (14–17).

While both manual and automated massage therapies show potential, there is currently no comprehensive synthesis comparing their effectiveness and safety. Understanding the benefits and limitations of each approach is especially important for the medical community to confidently implement these therapies.

Thus, this systematic review and meta-analysis evaluates the effectiveness and safety of colonic abdominal massage as an intervention for individuals with CC, including both functional and neurogenic forms. It synthesizes evidence from randomized controlled trials (RCTs), prospective comparative cohort studies and case series, covering children, adults, and elderly populations with diverse underlying conditions such as spinal cord injury, multiple sclerosis, and cerebral palsy. The review compares manual and automated massage techniques, assessing outcomes such as number of bowel movements per week (BM/w), QoL, and adverse events (AEs). This review aims to inform clinical decision-making and guide future research in nonpharmacological management of CC.

## METHODS

This systematic review was conducted in accordance with the Cochrane Handbook for Systematic Reviews and Preferred Reporting Items for Systematic reviews and Meta-Analyses (PRISMA) guidelines (18,19). The study protocol was registered with ClinicalTrials.gov (NCT06695754).

A literature search was conducted to identify studies (1990–2025) on manual and device-assisted abdominal colonic massage for constipation, bowel function, or symptom-related outcomes affecting QoL. Searches in PubMed, Google Scholar, Ovid, and Cochrane Central Register of Controlled Trials used MeSH keywords “colon, abdominal, bowel function, constipation, massage, device (see Supplementary Digital Content, http://links.lww.com/CTG/B494).”

Two reviewers independently screened title, abstracts, and full text, with a third resolving any disagreements. Included studies were peer-reviewed, available online, and involving manual or automated abdominal colonic massage for treating constipation. Excluded were studies targeting other conditions, reporting only incidence rates, or lacking preintervention/postintervention data (mean differences or standard deviations (SDs)) required for quantitative synthesis (Table 1).

**Table 1.** Inclusion and exclusion criteria

Criteria	Inclusion	Exclusion
Study type	Clinical studies using abdominal/colon massage to treat constipation	Reviews, conference abstracts, case reports, dissertations, registration documents, clinical experience summaries, animal experiments
Publication date	Published between 1990 and 2025	Published outside 1990 and 2025
Patient profile	Patients with constipation of any age, sex, and ethnicity	Non constipated subjects
Interventions	Manual or automated colon massage treatment for constipation	Other treatments for constipation such as surgical interventions, acupuncture, acupoint application, medicinal alternative therapies
Outcomes	Evacuation frequency, quality of life parameters, constipation symptoms	Other outcomes related to constipation symptoms and data in noncomparable formats (e.g. missing outcomes or statistical details, or data that cannot be standardized across studies)
Language	English, German, Spanish, Italian, French, Portuguese	Languages other than English, German, Spanish, Italian, French, Portuguese
Availability	Abstract available	No abstract available
Case data	Complete case data	Incomplete case data

Summary of eligibility criteria used for study selection, including study type, publication date, original work, patient profile, intervention, outcomes, language, and data availability.

Data extracted included the first author, publication year, sample size, participant characteristics (age, comorbidities), interventions and control details, treatment duration, study design, AEs reported, and outcome measures. Studies lacking standardized or comparable data were excluded to maintain the validity and reliability of the synthesis.

The outcome measures included at least one of the following: BM/w, QoL, symptoms of constipation, and AEs. The primary quantitative parameter was the change in evacuation frequency, calculated as the difference in BM/w (mean [SD]) after a period of colonic massage (POST) compared with baseline before massage (PRE). These values were either directly extracted from the original publications or calculated using the data provided. The difference between the POST and PRE in QoL was normalized to percentage change (%, mean [SD]) according to the formula: (Observed score/[Max-Min possible score]) × 100 to facilitate comparison across studies using different scoring instruments. For clarity, all QoL outcomes were expressed as improvements in health status. Where multiple scales on the same paper were available, the most used across studies was selected (see Supplementary Digital Content, http://links.lww.com/CTG/B494).

The incidence of AEs was expressed as percentage, detailing severity when reported.

Study quality was assessed using Cochrane-recommended tools (20,21). Risk of bias (RoB) was evaluated across 7 domains—randomization/confounding, exposure classification, participant selection, deviations from intended interventions, missing data, outcome measurement, and selective reporting—for the assessed outcomes across all intervention groups. Ratings were categorized as low risk, some concerns, high risk, or not applicable.

Data transformation and statistical analyses were conducted using Excel (Microsoft Office 365, Microsoft Co., 2024) and GraphPad Software Prism 5 for Windows (version 5.03, 2007; La Jolla, CA), following the ICH E9 “Statistical Principles for Clinical Trials” and according to previously published guidelines (22). All analyses were performed on the intention-to-treat (ITT) population for each study and across all assessed outcomes. Results were displayed as forest plots.

Meta-analyses were performed on RCTs and cohort studies (single-arm and double-arm) evaluating abdominal colonic massage interventions. Studies were grouped by intervention type (manual or device-assisted), stratified for patient pathology profile and analyzed separately for outcome. Standardized mean differences (SMDs) with 95% confidence intervals (CIs) were calculated using Cohen's *d*. For single-arm studies, SMDs reflected change at POST respect to PRE intervention, while for double-arm studies, they reflected the difference in POST-PRE between intervention and control groups. A random-effects model via I^2^ statistics was used to account for heterogeneity (23). Pooled effects were tested using Z statistics (*P* < 0.05). Publication bias and small-study effects were evaluated using the Egger test (*P* < 0.05) (24). To evaluate the potential impact of clinical heterogeneity and the robustness of the results, post hoc analyses were conducted considering patient's age and intervention's follow-up duration (see Supplementary Digital Content, http://links.lww.com/CTG/B494). Additional sensitivity analyses tested the exclusion of statistical outliers (Z > 1.96) (25).

To accurately estimate the effects of manual (MAN) and automated (AUTO) interventions and reduce potential bias, we conducted an additional statistical synthesis where control group data from original studies, when available, were extracted and treated as a third comparator (CONT) vs MAN and AUTO groups. Effect sizes were calculated as mean differences between POST- and PRE-intervention values and presented with 95% CI. Within-group effect sizes were assessed using paired *t*-test. Group differences were analyzed using 2-way analysis of variance (α = 0.05), with a Bonferroni post hoc test for pairwise comparisons. Continuous outcomes were reported as mean (SD). All *P*-values are reported to ensure transparency.

## RESULTS

**Figure 1.**
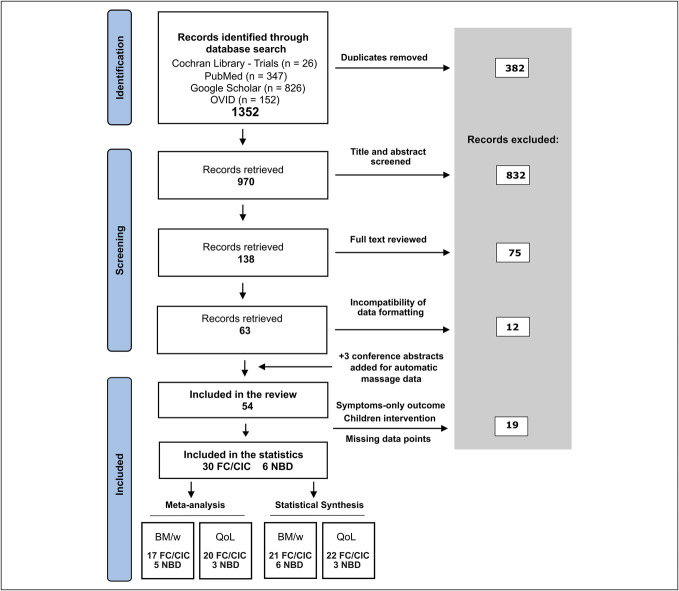
PRISMA flow diagram illustrating the study selection process. The figure details the number of records identified, screened, assessed for eligibility, and included in the systematic review. It reports the number of excluded records and the final number of studies contributing to the following predefined outcomes: BM/w and QoL. BM/w, bowel movements per week; CIC, chronic idiopathic constipation; FC, functional constipation; NBD, neurogenic bowel dysfunction; QoL, quality of life.

A total of 1,352 records were identified. After removal of duplicates and exclusion based on predefined criteria (Table 1), 54 studies (ITT: 3,113) were retained for qualitative and quantitative synthesis. Studies reporting only symptoms of constipation outcomes were excluded because heterogeneous, nonstandardized scales precluded direct comparison. Pediatric studies were excluded to homogenize the sample population. Additional exclusions were based on missing or incompatible data formats (Figure 1). This resulted in 35 publications (ITT: 1,965). Of these, 15 studies (ITT: 760) assessed the AEs; 26 (ITT: 1,407) contributed data on the primary outcome BM/w, and 23 (ITT: 1,658) contributed to the secondary outcome QoL. These numbers include 3 additional conference abstracts (26–28) (combined ITT: 36) (Figure 1). Most of the studies focused on functional constipation or chronic idiopathic constipation (FC/CIC, 85.7%, ITT: 1,672) followed by neurogenic bowel dysfunction (NBD, 17.1%, ITT: 293). Interventions included either manual (80.0%, ITT: 1,759) or automated massage therapies (20.0%, ITT: 206). Sample size ranged from 6 to 312 patients/study, aged 58.7 (17.4) year in average (18–90 year). In 71.4% of studies (ITT: 1,499), treatment duration ranged between 3 and 12 weeks with 65.7% of RCTs (ITT: 1,674). No study was judged to be at high RoB. Most studies were rated as low risk among all domains (Table 2).

**Table 2.** Overview of the included studies characteristics and corresponding risk of bias assessment

Reference	Intervention time	Study design	Massage modality	ITT	Patient pathol	DEAL with AE	Type of RoB (combined cochrane RoB 2 and the ROBINS-I tool)							
							1.A. RandP	1.B. Conf	2 ExpClas	3. PartSel	4 DevIn	5. MisDat	6 OutMeas	7 SelRep
Lämås et al (8)	8 wk	RCT	Manual	60	FC/CIC	No	SC	—	—	—	SC	LR	LR/SC	LR
McClurg et al (9)	6 wk	RCT	Manual	180	NBD	Yes	LR	—	—	—	LR	LR	LR	LR
Doǧan et al (11)	4 wk	RCT	Manual	74	FC/CIC	No	LR	—	—	—	LR	SC	LR/SC	LR
Birimoglu et al (12)	3 d	RCT	Manual	36	FC/CIC	No	LR	—	—	—	LR	LR	LR	LR
McClurg et al (13)	4 wk	RCT	Manual	30	FC/CIC	Yes	LR	—	—	—	LR	LR	LR/SC	LR
Mimidis et al (14)	6 wk	Cohort study	Automated	30	FC/CIC	Yes	—	SC	LR	LR	LR	LR	LR	LR
Choi et al (15)	2 wk	Cohort study	Automated	37	FC/CIC	Yes	—	HR	LR	LR	LR	SC	SC	SC
Bremer et al (16)	40–96 wk	Cohort study	Automated	11	NBD	Yes	—	SC	LR	LR	LR	LR	LR/SC	LR
McClurg et al (17)	4 wk	Cohort study	Automated	92	CIC, NBD	Yes	—	SC	LR	LR	LR	LR	LR/SC	LR
Herrero-Fresneda et al (26)	2 wk	Cohort study	Automated	18	FC/CIC	Yes	—	HR	LR	SC	LR	SC	LR/SC	SC
Herrero-Fresneda et al (27)	4 wk	Cohort study	Automated	8	FC/CIC	Yes	—	HR	LR	LR	LR	SC	LR/SC	LR
Herrero-Fresneda et al (28)	24 wk	Case series	Automated	10	NBD	Yes	—	SC	SC	SC	SC	LR	LR	LR
Klauser et al (29)	3 wk	C-cohort study	Manual	9	FC/CIC	No	—	HR	SC	HR	SC	HR	SC	SC
Tarsuslu et al (30)	6 wk	Cohort study	Manual	7	FC/CIC	No	—	HR	LR	SC	LR	LR	SC	SC
Bezgin et al (31)	6 wk	RCT	Manual	30	FC/CIC	No	SC	—	—	—	LR	LR	HR	LR
Chatip et al (32)	3 wk	RCT	Manual	20	NBD	No	SC	—	—	—	LR	LR	LR/SC	LR
Lämås et al (33)	8 wk	RCT	Manual	60	FC/CIC	No	SC	—	—	—	SC	LR	SC	LR
Albers et al (34)	3 wk	RCT	Manual	14	NBD	No	SC	—	—	—	LR	LR	LR	LR
Ayas et al (35)	3 wk	Cohort study	Manual	24	NBD	No	—	SC	LR	LR	LR	SC	LR	LR
Belvaux et al (36)	4 wk	Cohort study	Manual	21	FC/CIC	No	—	SC	LR	LR	LR	SC	LR/SC	LR
Brugman et al (37)	4 wk	Cohort study	Manual	6	FC/CIC	No	—	SC	LR	SC	LR	SC	SC	LR
Çetinkaya et al (38)	4 wk	RCT	Manual	60	FC/CIC	No	SC	—	—	—	SC	LR	SC	LR
Çevik et al (39)	4.3 wk	Cohort study	Manual	25	FC/CIC	No	—	SC	LR	LR	LR	SC	LR/SC	LR
Dadura et al (40)	8 wk	RCT	Manual	18	FC/CIC	Yes	SC	—	—	—	LR	LR	LR/SC	LR
Dehghan et al (41)	3 d	RCT	Manual	82	CIC	Yes	SC	—	—	—	LR	LR	LR	LR
Drouin et al (42)	4 wk	RCT	Manual	36	FC/CIC	No	LR	—	—	—	LR	LR	LR/SC	LR
Faghihi et al (43)	2 wk	RCT	Manual	60	FC/CIC	No	SC	—	—	—	LR	LR	SC	LR
Fekri et al (44)	1.4 wk	RCT	Manual	68	FC/CIC	No	SC	—	—	—	SC	LR	SC	SC
Güven et al (45)	6 wk	RCT	Manual	100	FC/CIC	No	LR	—	—	—	LR	LR	SC	LR
Hasanshahi et al (46)	4.7 wk	RCT	Manual	76	FC/CIC	No	LR	—	—	—	LR	LR	LR	LR
Karaaslan et al (47)	4 wk	RCT	Manual	44	FC/CIC	Yes	LR	—	—	—	LR	LR	SC	LR
Kassolik et al (48)	3 wk	RCT	Manual	28	CIC, IBS	Yes	LR	—	—	—	LR	LR	LR	LR
Lafcı et al (49)	3 wk	RCT	Manual	50	FC/CIC	No	SC	—	—	—	LR	LR	LR	LR
Li et al (50)	2 wk	C-cohort study	Manual	100	FC/CIC	Yes	—	SC	LR	LR	LR	LR	SC	SC
McClurg et al (51)	6 wk	RCT	Manual	32	NBD	No	LR	—	—	—	LR	LR	SC	LR
Mokhtare et al (52)	2 wk	RCT	Manual	104	FC/CIC	Yes	LR	—	—	—	LR	LR	LR/SC	LR
Nouhi et al (53)	3 d	RCT	Manual	66	FC/CIC	No	LR	—	—	—	LR	LR	SC	LR
Olgun et al (54)	8 wk	RCT	Manual	60	FC/CIC	No	LR	—	—	—	LR	LR	LR/SC	SC
Orhan et al (55)	4 wk	RCT	Manual	40	FC/CIC	No	LR	—	—	—	LR	LR	LR	LR
Seyedrasooli et al (56)	4 wk	RCT	Manual	40	FC/CIC	No	SC	—	—	—	LR	LR	LR	LR
Silva et al (57)	6 d	RCT	Manual	72	FC/CIC	No	LR	—	—	—	LR	LR	LR	LR
Turan et al et al (58)	6 mo	RCT	Manual	60	FC/CIC	Yes	SC	—	—	—	SC	LR	LR	LR
Yıldırım et al (59)	3 d	RCT	Manual	312	FC/CIC	No	LR	—	—	—	LR	LR	LR	LR
Assadollahzadeh et al (60)	8 wk	RCT	Manual	70	FC/CIC	No	SC	—	—	—	LR	LR	LR/SC	LR
Bai et al (61)	2 wk	RCT	Manual	196	FC/CIC	No	SC	—	—	—	LR	LR	SC	LR
Boangmanalu et al (62)	3 d	RCT	Manual	30	FC/CIC	No	SC	—	—	—	LR	LR	SC	LR
Daneshfar et al (63)	4 wk	RCT	Manual	77	FC/CIC	No	LR	—	—	—	LR	LR	LR	LR
Jafar et al (64)	4 wk	RCT	Manual	40	FC/CIC	No	SC	—	—	—	LR	LR	SC/HR	SC
Küçükaydınoğlu et al (65)	3 d	RCT	Manual	68	FC/CIC	No	LR	—	—	—	LR	LR	LR/SC	LR
Li et al (66)	4 wk	RCT	Manual	74	FC/CIC	No	LR	—	—	—	LR	LR	LR	LR
Malekiantaghi et al (67)	6 wk	RCT	Manual	66	FC/CIC	Yes	SC	—	—	—	LR	LR	LR	LR
Shi et al (68)	10 d	RCT	Manual	90	FC/CIC	Yes	LR	—	—	—	LR	LR	LR	LR
Suantika et al (69)	3 d	Cohort study	Manual	30	FC/CIC	No	—	HR	LR	LR	LR	LR	SC	LR
Wang et al (70)	4 wk	RCT	Manual	60	NBD	Yes	SC	—	—	—	LR	LR	LR	LR

RoB (20,21) was assessed across 7 domains (1A-RandP: randomization process, 1B-Conf: confounding, 2-ExpClas: exposure classification, 3-PartSel: participant selection, 4-DevIn: deviations from intended interventions, 5-MisDat: missing data, 6-OutMeas: outcome measurement, and 7-SelRep: selective reporting), categorized as LR, SC, HR; or — (not applicable).

AEs, adverse events; C-Cohort study, comparative double-arm cohort studies; CIC, chronic idiopathic constipation; FC, functional constipation; HR, high risk; IBS, irritable bowel syndrome; ITT, intention to treat; LR, low risk; NBD, neurogenic bowel dysfunction; RoB, risk of bias; RCT, randomized controlled trial; SC, some concerns.

### Adverse events

**Figure 2.**
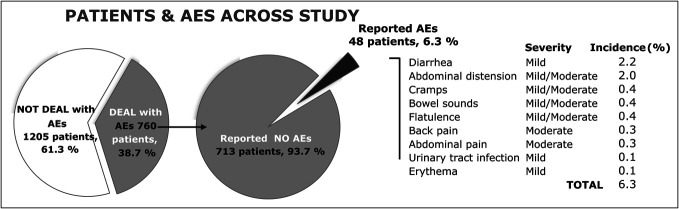
Distribution and characterization of AEs among patients included in the systematic review. The left pie chart illustrates the proportion of patients in studies that assessed AEs (DEAL with AEs) vs those that did not (NOT DEAL with AEs). The right pie chart focuses on the subset of patients from AE-investigated studies, showing the percentage and number of patients who experienced AEs (reported AEs), and those who did not report any side effects (reported NO AEs). Reported AEs are categorized by type and severity, including only events deemed related or possibly related to the intervention. Incidence was calculated as AE-cases over all patients in AE-assessed studies (n = 760). AE, adverse event.

All the automated and some manual studies (k = 15) explicitly reported AEs, with no AEs in most of them and only mild, self-limiting AEs affecting 48 patients, with an overall incidence of 6.3%. The other 20 studies—all involving manual interventions—did not deal with AEs (Figure 2).

### Meta-analysis results; heterogeneity, sensitivity analysis, and publication bias

Studies included in the meta-analysis of BM/w and QoL were 21 and 22, respectively (Figure 1). Twenty-two involved FC/CIC, 4 NBD, and 1 both patient profiles. In NBD patients, BM/w quantitative synthesis was limited by the small number of eligible studies (manual: k = 2; device-assisted: k = 3) and was not feasible with QoL outcomes. Manual studies included RCTs and cohort studies (single- and double-arm). Automated device-assisted included single-arm cohort studies.

### Primary outcome: evacuation frequency (BM/w)

**Figure 3.**
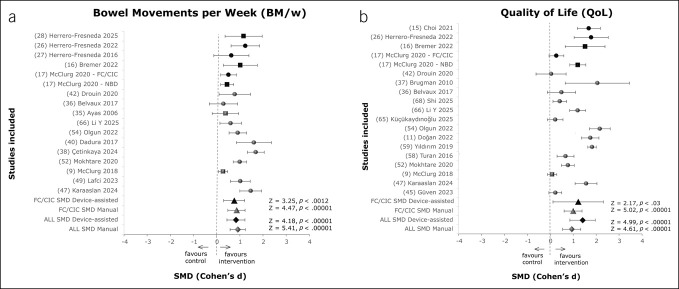
Meta-analysis forest plot of manual and device assisted interventions stratified by patient profiles. Results are displayed as SMDs (Cohen's *d*) with 95% CIs as (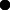) FC/CIC device-assisted (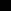); NBD device-assisted; (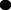) FC/CIC manual (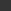); NBD manual. Pooled estimates of SMD are displayed as (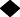) all device-assisted (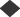); all manual (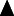); FC/CIC device-assisted, and (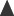) FC/CIC manual interventions. NBD results were not analyzed because of insufficient sample. The vertical dashed line indicates the null effect. Estimates were derived from RCTs and cohort studies using a random-effects model to account for between-study variability. Overall effect significance: Z test *P* < 0.05. The exact values are detailed in Table 3. CIC, chronic idiopathic constipation; FC, functional constipation; NBD, neurogenic bowel dysfunction; SMD, standardized mean difference.

The manual studies showed a significant improvement in BM/w (SMD = 1.45, *P* < 0.0001) with high heterogeneity I^2^ value and evidence of small-study effects (Figure 3a and Table 3). The device-assisted studies showed also improvements (SMD = 1.32, *P* = 0.0006), with slightly lower heterogeneity but no evidence of small-study effects. These findings remained robust even after excluding outliers in the sensitivity analyses. Further stratifying patients by pathology profile showed the same trend. Considering only FC/CIC patients, BM/w improved similarly in manual (SMD = 1.49, *P* < 0.0001) and device-assisted interventions (SMD = 1.50, *P =* 0.0091) keeping similar high heterogeneity, and showing evidence of small-study effects only in the manual interventions. In NBD patients, manual interventions yielded the lower effect (SMD = 0.29, *P =* 0.0025), whereas device-assisted therapies showed a higher effect (SMD = 0.76, *P =* 0.0022).

**Table 3.** Meta-analysis and sensitivity analysis comparing manual and device-assisted interventions across all studies (ALL) and within patient subgroups FC/CIC and NBD

Meta-analysis statistics			k (n)	Pooled ES	95% CI	Median ES	IQR 1–3	P ES	I^2^	P Egger's test	*P* value *t* test
BM/w	ALL	Manual	15	1.45	0.972–1.921	1.02	0.69–2.57	<0.0001	93.3	0.01	0.51
		Device-assisted	6	1.32	0.571–2.073	1.2	1.03–4.81	0.0006	89.2	0.08	
	FC/CIC	Manual	13	1.49	0.96–2.027	1.47	0.91–2.91	<0.0001	93.5	0.04	0.07
		Device-assisted	4	1.50	0.371–2.621	0.93	0.6–3.27	0.0091	92.2	0.57	
	NBD	Manual	2	0.29	0.102–0.476	0.33	0.3-	0.0025	0	—	—
		Device-assisted	3	0.76	0.274–1.254	1.02	0.74–1.16	0.0022	53.2	—	
	ALL (wo outliers)	Manual	11	0.91	0.578–1.235	0.91	0.49–1.47	<0.0001	85.9	0.21	0.92
		Device-assisted	5	0.83	0.441–1.218	1.2	1.03–4.06	<0.0001	56.3	0.06	
	FC/CIC (wo outliers)	Manual	9	0.85	0.479–1.227	1.0	0.78–1.54	<0.0001	87.1	0.69	0.25
		Device-assisted	3	0.75	0.299–1.209	0.63	0.57–1.24	0.0012	51.4	0.57	
QoL	ALL	Manual	18	1.57	1.045–2.098	1.09	0.43–2.08	<0.0001	97.2	0.06	0.29
		Device-assisted	4	1.41	0.854–1.958	1.6	1.36–1.76	<0.0001	76.7	0.12	
	FC/CIC	Manual	17	1.67	1.124–2.217	1.17	0.49–2.11	<0.0001	96.8	0.15	0.07
		Device-assisted	3	1.21	.0.118–2.312	1.68	0.97–1.78	0.03	93.1	0.30	
	NBD	Manual	1	—	—	—	—	—	—	—	—
		Device-assisted	2	1.24	0.918–1.566	1.36	1.28-	<0.0001	—	—	
	ALL (wo outliers)	Manual	15	0.93	0.534–1.325	0.77	0.316–1.74	<0.0001	95.1	0.87	0.24
		Device-assisted	4	1.41	0.854–1.958	1.6	1.36–1.76	<0.0001	76.7	0.12	
	FC/CIC (wo outliers)	Manual	14	0.99	0.605–1.381	0.88	0.43–1.76	<0.0001	93.7	0.54	0.06
		Device-assisted	3	1.21	0.118–2.312	1.68	0.97–.78	0.03	93.1	0.30	

Number of studies (k), pooled effect size (Pooled ES, Cohen's d), 95% CI, median effect size (Median ES), interquartile range of effect sizes (IQR 1–3), *P*-value for the pooled effect size (P ES, Z test), heterogeneity across studies (I^2^), *P*-value for Egger test (P Egger test), and *P*-value from *t* test comparisons (*P t* test) between manual and device-assisted outcomes across all studies (ALL) and cohorts involving FC/CIC or NBD patients only. Analyses excluding outliers are provided for ALL and FC/CIC groups. The Egger test evaluates small-study effects, and Z-tests assesses the overall significance of the pooled estimates.

CIC, chronic idiopathic constipation; ES, effect size; FC, functional constipation; NBD, neurogenic bowel dysfunction.

### Secondary outcome: QoL

Among all the analyzed studies, 68.5% used the PAC-QoL scale while the remaining 31.8% used other tools, each used in ≤5% of studies (see Supplementary Digital Content, http://links.lww.com/CTG/B494) (Figure 3b and Table 3).

Manual studies showed a strong positive effect (SMD = 1.57, *P* < 0.0001), with high heterogeneity though with no small-study effect. Similarly, device-assisted studies showed a significant improvement (SMD = 1.41, *P* < 0.0001), with lower heterogeneity and no evidence of small-study effect. For FC/CIC patients, manual intervention was associated with the higher effect (SMD = 1.67, *P* < 0.0001). Device-assisted studies showed a slightly lower effect (SMD = 1.21, *P* = 0.03) not statistically different from the manual intervention. Heterogeneity remained high in both strata, with no evidence of small-study effects. Sensitivity analysis for QoL also confirmed the result stability.

### Statistical synthesis

Control data (patients under sham or no treatment) from RCTs and double-arm cohort studies was extracted and compiled in a third group (CONT) compared with MAN and AUTO groups. Studies analysed for BM/w and QoL were 27 and 25, respectively (Figure 1), of which 21 involved FC/CIC, 5 NBD, and 1 both patient profiles.

### Primary outcome: evacuation frequency (BM/w)

**Figure 4.**
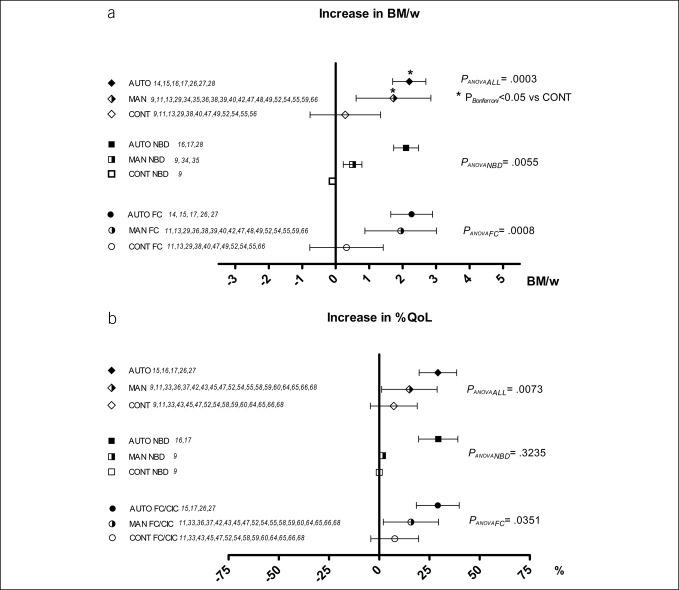
PRE-POST effect size of BM/w and QoL outcomes. Data are provided for FC/CIC and NBD cohorts separately and for all studies together (all). The effect sizes of each outcome are displayed as mean ± 95% CI. Evacuation frequency is shown as number of bowel movements per week (a). Quality of life (b) is shown as a percentage relative to each study's scale. Exact values appear in Table 4. The numbers beside each group symbol indicate the reference ID of the studies included in that analysis (i.e. Bremer et al, McClurg et al, and Herrero-Fresneda et al [16, 17, 28] [n = 3] in AUTO NBD group for QoL). *P* values from ANOVA, and Bonferroni tests. *p_*Bonferroni*_< 0.05 vs CONT. ANOVA, analysis of variance; AUTO, automated; BM/w, bowel movements per week; CIC, chronic idiopathic constipation; CONT, comparator; FC, functional constipation; MAN, manual; NBD, neurogenic bowel dysfunction; SMD, standardized mean difference; QoL, quality of life.

Evacuation frequency at baseline was comparable across all study groups (Figure 4a, Table 4). The improvement in BM/w at the end of the intervention was statistically significant (*P* < 0.05) for both MAN and AUTO when including all the studies (ALL) and compared to the CONT group. Effect size vs CONT was 5.9 and 7.6 times in MAN and AUTO, respectively. Pathology-specific stratification maintained the relative effect-size profile in FC/CIC, while NBD patients showed MAN and AUTO effect sizes of 5 and >20 times that of the CONT group. Either case, no significant differences were found between AUTO and MAN interventions.

**Table 4.** Statistical synthesis: comparison between automated, manual, and control groups

Outcome	Group _patient profile_	Group _treatment_	N	PRE	POST	*P*_POST-PRE_ paired *t* test	Effect size	*P* ANOVA
BM/w	ALL	AUTO	7	3.28 (1.25)	5.47 (1.10)	<0.0001	2.20 (1.74; 2.65)^a^	0.0003
		MAN	20	3.09 (0.88)	4.810 (1.20)	<0.0001	1.71 (1.18; 2.24)^a^	
		CONT	12	3.15 (1.68)	3.44 (1.27)	0.3645	0.29 (−0.38; 0.96)	
	NBD	AUTO	3	3.64 (1.40)	5.73 (1.47)	0.0105	2.09 (1.16; 3.02)	0.0055
		MAN	3	3.90 (0.11)	4.40 (0.18)	0.0894	0.50 (−0.19; 1.20)	
		CONT	1	4.00 (0.00)	3.90 (0.00)	NA	−0.1 (NA)	
	FC/CIC	AUTO	4	3.00 (1.25)	5.28 (0.94)	0.0050	2.28 (1.30; 3.25)	0.0008
		MAN	17	2.94 (0.88)	4.87 (1.29)	<0.0001	1.92 (1.36; 2.48)	
		CONT	11	3.07 (1.74)	3.39 (1.33)	0.3521	0.32 (−0.41; 1.06)	
QoL	ALL	AUTO	6	29.17 (14.45)	58.83 (7.06)	0.0004	29.67 (20.63; 38.70)	0.0073
		MAN	19	41.13 (30.77)	58.40 (24.66)	0.0001	17.27 (9.78; 24.76)	
		CONT	16	44.11 (27.34)	51.32 (24.20)	0.0258	7.21 (0.99; 13.43)	
	NBD	AUTO	2	33.59 (19.68)	63.00 (9.90)	0.147	29.42 (−58.45; 117.3)	0.3235
		MAN	1	60.00 (0.00)	61.67 (0.00)	NA	1.67 (NA)	
		CONT	1	55.42	55.42	NA	0.00 (NA)	
	FC/CIC	AUTO	4	26.96 (14.12)	56.75 (5.74)	0.0084	29.79 (14.56; 45.02)	0.0351
		MAN	18	40.08 (31.31)	58.22 (25.37)	0.0001	18.14 (10.42; 25.85)	
		CONT	15	43.36 (28.12)	51.05 (25.03)	0.0253	7.69 (1.10; 14.29)	

Data were stratified and analyzed according to patient profile (FC/CIC or NBD) and for all the retrieved studies (ALL). Values at baseline (PRE) and after intervention (POST) are shown as mean (SD) for the primary outcome BM/w, and for QoL. Effect size, defined as the difference between POST and PRE according to the paired *t* test, is shown as mean (5% CI; 95% CI). A positive value indicates an increase. Comparison among the effect size of each group (AUTO, MAN, and CONT) was made by ANOVA followed by Bonferroni test to compare each pair of groups.

ANOVA, analysis of variance; AUTO, automated; BM/w, bowel movements per week; CIC, chronic idiopathic constipation; CONT, comparator; FC, functional constipation; MAN, manual; NBD, neurogenic bowel dysfunction; QoL, quality of life.

a *P* < 0.05 vs CONT.

### Secondary outcomes: QoL

Baseline values for QoL were comparable across all study groups (Figure 4b, Table 4). MAN and AUTO interventions improved QoL vs CONT by 2.4 and 4.1 times, respectively, when including ALL studies. Stratified analyses confirmed the FC/CIC trend, and despite limited NBD studies, they showed no effect in CONT, while AUTO and MAN exhibited superior relative effects. Again, no significant differences were observed between the 2 intervention modalities.

## DISCUSSION

This systematic review with meta-analysis, consolidates clinical evidence on abdominal colonic massage, a safe, noninvasive treatment for CC.

Unlike pharmacological or invasive therapies, which often lose efficacy over time and cause adverse side effects (71), massage shows sustained benefits with continued use (16,17). Besides its benefits, manual massage presents challenges for long-term home use because of the need for daily application with proper technique. Even with standardized protocols, consistent execution is difficult to ensure—particularly for individuals with limited mobility, dexterity, or without caregiver support—making success heavily dependent on access to trained personnel and hence resulting in high cumulative costs (8,9,13,33,72).

Automated massage devices address these limitations by providing consistent, patient-controlled therapy at home, reducing reliance on caregivers and lowering long-term costs (33,72). Their ease of use supports daily adherence and integration into routine, helping establish a consistent, physiological bowel movement pattern. As a scalable solution, they represent a potentially superior alternative to manual techniques.

Therefore, comparing manual and automated massage was essential to determine whether automation can overcome usability barriers while preserving clinical efficacy and safety, enabling confident adoption into standard care.

Given the limited number of studies specifically examining automated massage—a central focus of this review—we supplemented our dataset with 3 additional studies (26–28). Although such abstracts fall outside the inclusion criteria, their directed relevance to the comparison with manual massage justified their inclusion. This approach represented our only viable method to slightly increase the sample size of the AUTO group, enabling a meaningful comparative analysis.

Among the various outcomes assessed, only evacuation frequency—a quantitative and objective measure—was consistently reported across most of the studies and was therefore selected as the primary outcome in this study. QoL and constipation symptoms were also evaluated, but the use of diverse scales hindered direct comparison. To address this, we harmonized scores into percentages, as recommended (73). This allowed for comparability among QoL measures, mostly based on PAC-QoL and similar instruments, but not for constipation symptoms, where scale variability introduced bias and prevented meaningful comparison. Therefore, symptoms-only reporting papers were excluded from the meta-analysis.

Although the meta-analysis can include single-arm cohort studies (19), comparing them with double-arm studies may introduce some bias because only manual interventions had control groups. To address this, we analyzed manual interventions separately from their controls, grouping the latter into a third, independent control group (CONT) for comparison with both manual (MAN) and automated (AUTO) interventions. Effect size in each group was defined as the “POST-PRE” difference and expressed in the same units than the analyzed outcome—BM/w and % of QoL improvement—to facilitate interpretation (73). This approach enabled fair comparison across the 3 groups, allowing inclusion of both comparative and noncomparative studies, and enhancing confidence in effect estimates (74). It also allowed the inclusion of studies excluded from the meta-analysis because of missing SD values, thereby broadening the analytical scope.

The meta-analysis showed that both manual and automated abdominal massage interventions were associated with meaningful improvements in evacuation frequency and QoL. These effects remained robust in stratified analysis by patient pathological profile. Meta-analyses in FC/CIC confirmed significant improvements in evacuation frequency and QoL across intervention types. In NBD, pooled estimates suggested modest gains in BM/w and directionally favorable effects on QoL, despite the few eligible studies. These findings strengthen the existing evidence (10,75–77) that supports abdominal massage as a beneficial intervention for improving gastrointestinal function and patient well-being, with effects that seem robust across different study designs and populations.

These results were reinforced by the statistical synthesis through the analysis of variance comparison among the 3 groups and the 2 patient profiles FC/CIC and NBD. PRE-POST effect sizes of BM/w in the AUTO group were comparable with those in the MAN group and both clearly higher than those observed in the CONT group. Comparable means and effect sizes were observed in the FC/CIC cohort. The apparent lower effect of manual intervention on NBD remains inconclusive given that it is supported by only 3 studies. Regarding QoL, the PRE-POST effect sizes were also higher than CONT, although here, the differences reached no statistical significance. Similarly, when the 2 patient cohorts were analyzed separately, the differences between either intervention and the control group did not reach statistical significance for either BM/w or QoL. This is most likely due to the small sample size, either in the AUTO group despite having added the above-mentioned additional studies, and in the patient cohorts, especially in the NBD 1. Nevertheless, as widely acknowledged (78–80) lack of statistical significance does not imply lack of clinical relevance: Patients in the AUTO groups (FC/CIC, NBD, and ALL) had lower baseline QoL and showed greater improvement by the end of treatment.

The likelihood that abdominal massage, either manual or automated, benefits both NBD and FC/CIC stems from the dominant role of the enteric nervous system (ENS) in colonic motility. ENS driven intrinsic reflexes coordinate peristaltic and mass movements largely independently of central or autonomic inputs, allowing key mechanisms of abdominal massage—mechanical mobilization of feces, increased blood flow and secretion, and activation of somato-visceral reflexes—to remain effective even when extrinsic innervation is impaired. Although conditions such as multiple sclerosis and Parkinson disease may affect the ENS, the pathways through which massage modulates motility are largely preserved. In the proximal colon, effects likely involve somato-autonomic activation through vagal pathways, whereas the more autonomously regulated distal colon responds mainly through local ENS mediated somato-visceral mechanisms (81). This dual-pathway model provides a coherent physiological basis for similar therapeutic responses in both NBD and FC/CIC. Despite its physiological plausibility, this mechanistic framework remains hypothetical and requires empirical verification.

Other published systematic reviews have shown the effectiveness of the manual massage on CC (75–77); however, AEs were not analyzed. This review assessed AEs, whose reporting varied across studies. More than half of the studies, all involving manual interventions, did not mention AEs. This omission may reflect underreporting, not necessarily absence of AEs. However, owing to the low risk-profile of the intervention, it would be reasonable to assume no serious AEs and self-remitting AEs in those not-reporting studies. Future studies should provide more consistent safety reporting, explicitly documenting both the presence and absence of AEs to minimize reporting bias and strengthening the overall safety profile of these manual techniques. All studies involving automated massage reported on AEs, which were rare, mild, and self-limiting. Therefore, the high percentage of no AEs reported together with the very low incidence and severity of the reported ones, suggest that massage therapies, either manual or automated, are safe and well-tolerated.

Unlike previous studies (75–77), here not only RCTs were analyzed but also single-arm cohort studies and case series. This inclusive approach increased heterogeneity in patient profile and intervention follow-up, which can be seen as a limitation of the study. Another limitation is the small number of studies assessing automated interventions, which hindered the detection of statistical differences between intervention and control groups. As the sample was relatively homogeneous—adults with mainly FC—and the mechanisms of action of the abdominal colonic massage are likely similar across subpopulations (9,82), data pooling was justified (83,84). Stratified analyses of FC/CIC and NBD patients corroborated the full-dataset results, underscoring their robustness. The substantial variability observed in the manual massage group persisted despite sensitivity analyses, indicating that heterogeneity was inherent to the dataset rather than driven by outliers. Of note, even with lower sample size, the automated interventions showed smaller dispersion measures than the manual treatment, demonstrating the reproducibility and consistency of the device-assisted alternative in front of the high variability associated to manual massage. Nevertheless, complementary sensitivity and post hoc analyses accounting for patient age and follow-up duration (see Supplementary Digital Content, http://links.lww.com/CTG/B494), confirmed the stability and completeness of the results.

The study also has notable strengths. In line with COCHRANE guidelines (18,19) and Mathes et al (74), the inclusion of diverse study designs enhanced both sample size and data representativeness. Despite heterogeneity, findings consistently showed improved evacuation frequency and better QoL after colonic massage. The robustness of the results was further confirmed by low RoB and high consistency, even when outlier studies were excluded.

As noted in discussions of clinical vs statistical significance (80), consistent findings across diverse study designs, settings, and populations strengthen evidence of a true effect, supporting the reliability and clinical relevance of colonic massage as an effective intervention for CC.

Although more RCTs are needed to further validate the efficacy of device-assisted massage, current evidence indicates that it is as effective as the manual intervention and may even surpass it. Automated massage offers reproducible and scalable treatment, easing integration into daily care. Suitable for long-term use across settings, it supports efficient care delivery with minimal training, benefiting both patients and health care systems.

This systematic review synthesizes comparative and noncomparative studies evaluating manual or automated abdominal colonic massage on adult people suffering from either FC/CIC or NBD constipation. Both interventions showed very low, mild, self-limiting AEs and resulted in higher evacuation frequency and better QoL compared with controls, both in FC/CIC and NBD patients. As a nondrug noninvasive solution, abdominal colonic massage should be a reliable option to try before escalating in the bowel management pyramid (5).

While further RCTs on automated massages are warranted, current evidence suggests it may be as effective, or even superior, to manual techniques. In addition to its clinical benefits, automated massage offers consistent reproducibility and ease of daily integration, which are key factors for confidently incorporating it into standard care pathways like most European countries already do (85–91). Moreover, its scalability may help reduce the burden on health care systems.

## CONFLICTS OF INTEREST

**Guarantor of the article:** M. Eugenia Delgado, PhD.

**Specific author contributions:** M.E.D.: conducted the study; collected and interpreted data; drafted and approved the final draft submitted. J.B.: interpreted data; drafted, reviewed, and approved the final draft submitted. I.H.-F.: planned the study, reviewed and approved the final draft submitted.

**Financial support:** This work was supported by the European Union (NextGenerationEU) via the Spanish Government, Min. of Science and Innovation, and the State Research Agency (project CPP2022-009839).

**Potential competing interests:** M.E.D. and J.B. declare no conflict of interest. I.H.F. is an employee of usMIMA S.L., manufacturer of 1 of the devices evaluated as part of the automatic device-assisted intervention group.

**Ethics approval statement:** No patient consent nor ethics committee approval were need. The study was conducted following the Good Clinical Practice (GCP) and reported in accordance with the COCHRANE and PRISMA guidelines.

**Clinical trial registration:** This study is registered in the public trial registry ClinicalTrials.Gov under ID NCT066957.

## Supplementary Material

## ABBREVIATIONS:

AEs: adverse events
AUTO: automatic
BM/w: bowel movements per week
CC: chronic constipation
CI: confidence interval
CIC: chronic idiopathic constipation
CONT: control
ENS: enteric nervous system
FC: functional constipation
GCP: good clinical practice
HR: high risk
IBS: irritable bowel syndrome
ITT: intention-to-treat
LR: low risk
MAN: manual
NBD: neurogenic bowel dysfunction
PRISMA: Preferred Reporting Items for Systematic reviews and Meta-Analyses
QoL: quality of life
RCTs: randomized controlled trials
RoB: risk of bias
SC: some concerns
SD: standard deviation
SMD: standardazed mean differences
