# Supplementary material for: Safety and Efficacy of Manual and Automated Abdominal Colonic Massage for Chronic Constipation: A Systematic Review and Meta-Analysis
Source: Clin Transl Gastroenterol. 2026 Apr 1;17(7):e01027. doi: 10.14309/ctg.0000000000001027 (PMC13400043; doi:10.14309/ctg.0000000000001027)
Supplement: Supplementary file 1 [file ct9-17-e01027-s001.docx]

**Suplementary Digital Content**

**SDC1** -**Comprehensive electronic search strategies used across all databases included in the systematic review.** Searches carried out targeting studies published from January 1990 to December 2025 and the number of retrieved publications in each case. Search terms were tailored to each database’s indexing system and syntax, combining controlled vocabulary (e.g., MeSH) with relevant free-text keywords and free-text terms related to constipation, abdominal/colon massage, bowel function, transit time, and device use.

| Database | Search Strategy | Retrieved publications |
| --- | --- | --- |
| Cochrane Central Register of Controlled Trials  (Trials only) | constipation [MeSH Terms] AND (colon OR abdominal) AND massage | 2 |
|  | constipation AND "bowel function" AND (colon OR abdominal) AND massage | 11 |
|  | constipation AND colon AND ("bowel function" OR "transit time") AND "device" | 13 |
| PubMed (MEDLINE via NCBI) | constipation [MeSH Terms] AND (colon OR abdominal) AND massage | 134 |
|  | ("bowel function" OR "transit time") AND (colon OR abdominal) AND massage | 19 |
|  | constipation AND (colon OR abdominal) AND "device" | 129 |
|  | ("bowel function" OR "transit time") AND (colon OR abdominal) AND "device" | 65 |
| OVID (MEDLINE, Embase, AMED) (first 10 pages) | (PublicationDateRange:["1990-01-01T00:00:00Z" TO "2024-10-01T00:00:00Z"]) AND (Title:constipation) AND (AllFields:massage) AND (AllFields:abdominal) OR (AllFields:colon) | 141 |
|  | (PublicationDateRange:["1990-01-01T00:00:00Z" TO "2024-10-01T00:00:00Z"]) AND (Title:constipation) AND (Title:"device") AND (AllFields:abdominal) OR (AllFields:colon) OR (AllFields:"bowel function") OR (AllFields:" transit time") | 11 |
| Google Scholar (first 10 pages sorted by relevance) | constipation and (colon or abdominal) and massage | 211 |
|  | constipation and (colon or abdominal) and "device" | 227 |
|  | ("bowel function" or "transit time") and (colon or abdominal) and massage | 170 |
|  | ("bowel function" or "transit time") and (colon or abdominal) and "device" | 218 |

**SDC2**- **Scoring scales for quality-of-life assessment.** This table summarizes validated instruments used across included studies, listed alphabetically by first author publication. For each scale, the total number of items, scoring range (minimum to maximum), direction of improvement and original citation are provided.

| Scale | Total Items | Score (min-max) | QoL Interpretation | Orignal Ref. | Studies using scale |
| --- | --- | --- | --- | --- | --- |
| *EQ-5D-Visual Analogue Scale* (EuroQol-5 Dimension- VAS) | 5 | 0-100 | Improves with **higher** scores | Brooks R. EuroQol. PsycTESTS Dataset. 2016. | Ref. (34) |
| *WHOQOL-BREF-TR* (World Health Organization Quality of Life - BREF Turkish Version) | 27 | 0-100 | Improves with **higher** scores | Eser E, et al. Psychometric properties of the WHOQOL-100 and WHOQOL-BREF. 3P Dergisi. 1999 Jan 1;7:23–40. | Ref. (31) |
| *Modified PAC-QOL* | *28* | 28-140 | Improves with **lower** scores |  | Ref. (65, 12) |
| *PAC-QOL* (Patient Assessment of Constipation Quality of Life) | 28 | 0-112 | Improves with **lower** scores | Marquis P, et al. Development and validation of the Patient Assessment of Constipation Quality of Life questionnaire. Scand J Gastroenterol [Internet]. 2005;40(5):540–51 | Ref. (17, 56, 57,11, 63, 67, 72, 74, 75, 78, 79, 80, 84, 86, 88) |
| *NBIS* *QoL* (National Background Investigation Services Quality of Life) | 12 | 0-24 | Improves with **lower** scores | NBIS Group. National Background Investigation Services Quality of Life Questionnaire (NBIS QoL). 2015. | Ref. (9) |
| *STAI* (State-Trait Anxiety Inventory) | 20 | 20-80 | Improves with **lower** scores | Spielberger CD, et al. Manual for the State-Trait Anxiety Inventory. Palo Alto, CA: Consulting Psychologists Press; 1970 | Ref. (62) |
| *GCS* (General Comfort Scale) | 48 | 48-192 | Improves with **higher** scores | Kolcaba KY. Holistic comfort: operationalizing the construct as a nurse-sensitive outcome. ANS Adv Nurs Sci. 1992 Sep;15(1):1-10. | Ref. (85) |
| *Different VAS* (Visual Analogue Scale) | 1 | 1-6 | Improves with **lower** scores |  | Ref. (26, 16) |
|  | 1 | 0-100 | Improves with **higher** scores |  | Ref. (15) |

**SDC3**- **Robustness Testing of BM/W and QoL Outcomes for Manual and Device‑Assisted Colon Massage in ALL and FC/CIC Patient Groups.** Post‑hoc analyses summarizing alternative study combinations, reporting the number of contributing studies (k), pooled effect sizes (ES; Cohen’s d), 95% CI, median ES, IQR, heterogeneity (I²), Z‑test and Egger’s p‑values, and t‑test comparisons. Results are presented for ALL and FC/CIC subsets, with additional exclusions for follow‑up <2 weeks, inclusion of children, or both.

| **Post-hoc Meta-analysis** | | | | **k (n)** | **Pooled ES** | **95% CI** | **Median ES** | **IQR 1-3** | **P ES** | **I^2^** | **P Egger’s test** | **P t test** |
| --- | --- | --- | --- | --- | --- | --- | --- | --- | --- | --- | --- | --- |
| **BM/w** | **ALL Inc. Children** | **Manual** | | 17 | 1.48 | 1.036-1.924 | 1.02 | 0.61-2.91 | <0.0001 | 93 | 0.003 | 0.51 |
|  |  | **Device-Assisted** | | 6 | 1.32 | 0.571-2.073 | 1.2 | 1.03-4.81 | 0.0006 | 89.2 | 0.08 |  |
|  | **ALL Excl. <2weeks** | **Manual** | | 15 | 1.45 | 0.972-1.921 | 1.02 | 0.69-2.57 | <0.0001 | 93.3 | 0.01 | 0.46 |
|  |  | **Device-Assisted** | | 6 | 1.32 | 0.571-2.073 | 1.09 | 0.73-1.92 | 0.0006 | 89.2 | 0.075 |  |
|  | **ALL Incl. Children Excl. <2weeks** | **Manual** | | 17 | 1.48 | 1.036-1.924 | 1.02 | 0.61-2.91 | <0.0001 | 93 | 0.003 | 0.51 |
|  |  | **Device-Assisted** | | 6 | 1.32 | 0.571-2.073 | 1.2 | 1.03-4.81 | 0.0006 | 89.2 | 0.075 |  |
|  | **FC/CIC Inc. Children** | **Manual** | | 13 | 1.49 | 0.96-2.027 | 1.47 | 0.91-2.91 | <0.0001 | 93.5 | 0.04 | 0.07 |
|  |  | **Device-Assisted** | | 4 | 1.50 | 0.371-2.621 | 0.93 | 0.6-3.27 | 0.0091 | 92.2 | 0.57 |  |
|  | **FC/CIC Exc. <2weeks** | **Manual** | | 13 | 1.49 | 0.96-2.027 | 1.47 | 0.91-2.91 | <0.0001 | 93.5 | 0.038 | 0.06 |
|  |  | **Device-Assisted** | | 4 | 1.50 | 0.371-2.621 | 0.93 | 0.6-3.27 | 0.0091 | 92.2 | 0.571 |  |
|  | **FC/CIC Incl. Children Excl. <2weeks** | **Manual** | | 13 | 1.49 | 0.96-2.027 | 1.47 | 0.91-2.91 | <0.0001 | 93.5 | 0.04 | 0.07 |
|  |  | **Device-Assisted** | | 4 | 1.50 | 0.371-2.621 | 0.93 | 0.6-3.27 | 0.0091 | 92.2 | 0.57 |  |
| **QoL** | **ALL Inc. Children** | **Manual** | | 19 | 1.57 | 1.06-2.074 | 1.19 | 0.45-2.05 | <0.0001 | 97.0 | 0.055 | 0.29 |
|  |  | **Device-Assisted** | | 4 | 1.41 | 0.854-1.958 | 1.6 | 1.36-1.76 | <0.0001 | 76.7 | 0.116 |  |
|  | **ALL Excl. <2weeks** | **Manual** | | 15 | 1.86 | 1.231-2.492 | 1.56 | 0.63-2.16 | <0.0001 | 97.5 | 0.068 | 0.22 |
|  |  | **Device-Assisted** | | 4 | 1.41 | 0.854-1.958 | 1.6 | 1.36-1.76 | <0.0001 | 76.7 | 0.116 |  |
|  | **ALL Incl. Children Excl. <2weeks** | **Manual** | | 17 | 1.73 | 1.167-2.291 | 1.54 | 0.49-2.11 | <0.0001 | 97.2 | 0.032 | 0.26 |
|  |  | **Device-Assisted** | | 4 | 1.41 | 0.854-1.958 | 1.6 | 1.36-1.76 | <0.0001 | 76.7 | 0.116 |  |
|  | **FC/CIC Inc. Children** | **Manual** | | 17 | 1.67 | 1.124-2.217 | 1.17 | 0.49-2.11 | <0.0001 | 96.8 | 0.15 | 0.07 |
|  |  | **Device-Assisted** | | 3 | 1.21 | 0.118-2.312 | 1.68 | 0.97-1.78 | 0.03 | 93.1 | 0.3 |  |
|  | **FC/CIC Exc. <2weeks** | **Manual** | | 15 | 1.87 | 1.261-2.476 | 1.56 | 0.63-2.16 | <0.0001 | 96.9 | 0.14 | 0.06 |
|  |  | **Device-Assisted** | | 3 | 1.21 | 0.118-2.312 | 1.68 | 0.97-1.78 | 0.03 | 93.1 | 0.3 |  |
|  | **FC/CIC Incl. Children Excl. <2weeks** | | **Manual** | 14 | 2.00 | 1.35-2.641 | 1.65 | 0.82-2.61 | <0.0001 | 96.9 | 0.16 | 0.06 |
|  |  |  | **Device-Assisted** | 3 | 1.21 | 0.118-2.312 | 1.68 | 0.97-1.78 | 0.03 | 93.1 | 0.3 |  |
